# Non-invasive evaluation of bladder outlet obstruction in benign prostatic hyperplasia: a clinical correlation study

**DOI:** 10.1080/2090598X.2019.1660071

**Published:** 2019-09-09

**Authors:** S.V. Krishna Reddy, Ahammad Basha Shaik

**Affiliations:** a Department of Urology, Narayana Medical College, Nellore, India; b Department of Community Medicine, Narayana Medical College, Nellore, India

**Keywords:** Intravesical protrusion, prostate, lower urinary tract symptoms, bladder outlet obstruction, International Prostate Symptoms Score (IPSS), Transurethral Resection Of The Prostate (TURP)

## Abstract

**Objectives**: To determine the utility of ultrasonography (US)-derived parameters (e.g. prostate volume [PV], bladder wall thickness [BWT], post-void residual urine volume [PVR], and intravesical prostatic protrusion [IPP]) and uroflowmetry for identifying bladder outlet obstruction (BOO) by correlating them with the results of pressure–flow urodynamic studies (UDS).

**Patients and methods**: In all, 164 patients presenting with lower urinary tract symptoms suggestive of benign prostatic hyperplasia (BPH), from May 2016 to December 2018, were included in this study. All had International Prostate Symptoms Score (IPSS), Quality-of-Life (QOL) index, uroflowmetry (including maximum urinary flow rate [Q_max_]) and PVR measured by transabdominal US. Pressure–flow UDS were performed on all men and BOO was defined by a BOO Index (BOOI) >40. Men with a Q_max_ of ≥12.0 mL/s were considered to have ‘good’ flow.

**Results**: Amongst the 164 men, the mean (SD) age, PV, BWT and Q_max_ were 66.72 (9.88) years, 51.91 (13.24) mm, 5.07 (0.91) mm, and 8.46 (3.59) mL/s, respectively. In all, 91 (55.49%) patients had BOO with a BOOI >40 and nine (5.49%) had equivocal BOO with a BOOI of 20–40. The IPP was a statistically significant predictor (*P* < 0.001) of BOO compared with other variables in the initial evaluation. In patients with BOO confirmed by the pressure–flow UDS, IPP Grade III was associated with a higher BOOI than was Grade I and II (*P* < 0.001).

**Conclusion**: BWT, PV and PVR in conjunction with IPP are good predictors of clinically significant BOO due to BPH.

**Abbreviations**: AUC: area under the curve; BOOI: BOO Index; BPO, benign prostatic obstruction; BWT, bladder wall thickness; IPP: intravesical prostatic protrusion; P_det_: detrusor pressure; PV: prostate volume; PVR: post-void residual urine volume; Q_max_: maximum urinary flow rate; QOL: quality of life; ROC: receiver operating characteristic; (TA)US: (transabdominal) ultrasonography; UDS: urodynamic studies

## Introduction

BPH is a common benign disease of the prostate in ageing men. Prevalence of histological BPH increases with age, rising from ~40% in men aged 51–60 years to 90% by 81–90 years [1]. Symptoms of BPH often result from benign prostatic obstruction (BPO) associated with benign prostatic enlargement. But the symptoms and obstruction do not entirely depend on the prostate’s size. An assessment of clinically significant BOO has been done using different parameters. To date, most of the evaluations have focussed mainly on the presence of voiding dysfunction rather than the cause. Clinically significant BOO is urodynamically characterised by increased detrusor pressure (P_det_) and a decreased urinary flow rate [2]. Invasive urodynamic studies (UDS) testing for P_det_ are not routinely done in all patients with BPH. Non-invasive methods to diagnose BOO include: symptom evaluation (IPSS), PSA measurement, ultrasonography (US)-derived parameters such as prostate volume (PV), bladder wall thickness (BWT), intravesical prostatic protrusion (IPP), and post-void residual urine volume (PVR) [3,4]. Of these, US estimation of the prostate size and PVR with uroflowmetry have been routinely used by most urologists the world over to determine the presence of BOO. The sensitivity and specificity for BOO with maximum urinary flow rate (Q_max_) has limited value depending on the threshold used [5]. In contrast, IPP has been found to correlate with BOO [6]. IPP is a morphological change due to overgrowth of the prostatic median and lateral lobes into the bladder, and may lead to LUTS. The measurement of IPP is done by the vertical distance from the tip of the protruding prostate to the base of the bladder at the base of the prostate gland. IPP can be measured accurately and non-invasively by TRUS and can predict voiding parameters for determining BOO in men who present with LUTS. Ultrastructural changes in the bladder wall have been studied by Elbadawi et al. [7] from specimens obtained from patients with BOO and they found that there was an increased smooth muscle bulk, with or without interstitial collagen deposition. Therefore, it has been assumed that the measurement of this increase in BWT might be an indicator of the presence of BOO.

The main aim of the present study was to determine the utility of US-derived parameters (e.g. PV, BWT, and IPP) and uroflowmetry for identifying BOO, by correlating them with the results of pressure–flow UDS.

## Patients and methods

The study design was a prospective clinical research study, which was conducted in 172 consecutive men (aged >50 years) presenting with LUTS suggestive of BPH, from May 2016 to December 2018. Institutional ethics clearance and approval of the research study protocol was obtained prior to the study. Written informed consent was taken from all patients for photographing, recording and also its use for scientific and medical education purposes. The protocol, concept, design, and intellectual content for the study was drafted, conceived, and contributed by the first author who was blinded to relevant patient data at the time of its interpretation and statistical analysis. The patient data as per protocol was recorded by the second author. As per our protocol, our entry criteria included all symptomatic patients with LUTS due to clinically diagnosed BPH. The IPP is correlated with the severity of BOO due to BPH as assessed by symptoms score (IPSS), Quality-of-Life (QOL) index, uroflowmetry, and cystoscopy, and confirmed by a pressure–flow UDS. Those patients who were fit and willing for surgery underwent conventional TURP. Eight patients were excluded from the study. The exclusion criteria included: patients with BOO due to causes other than BPH; patients with prior urological surgery, such as TURP; open prostatectomy; diagnosed adenocarcinoma of prostate; patients with BPH with neurogenic bladder, urethral stricture, or vesical stones; and patients previously on medication (α-blockers, 5α-reductase inhibitors, anticholinergics, etc.) that may affect urine storage/voiding functions. In all patients, urine analysis and culture were done and those with positive cultures were treated with appropriate antibiotics before proceeding with the protocol. Renal function was assessed in all patients and those with renal insufficiency were excluded. The PSA level was also measured and those who had high values were excluded from the study.

**Figure 1.**
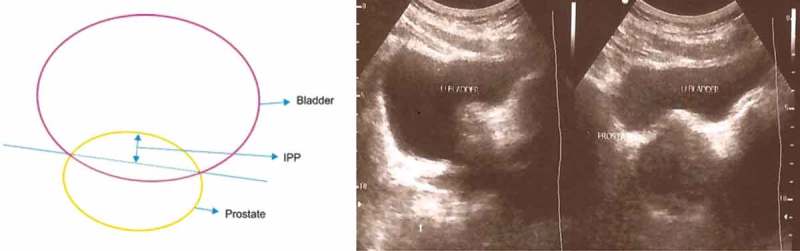
The vertical distance from the tip of the protrusion to the base of bladder was measured; longitudinal and sagittal views of the bladder and prostate using TAUS.

**Figure 2.**
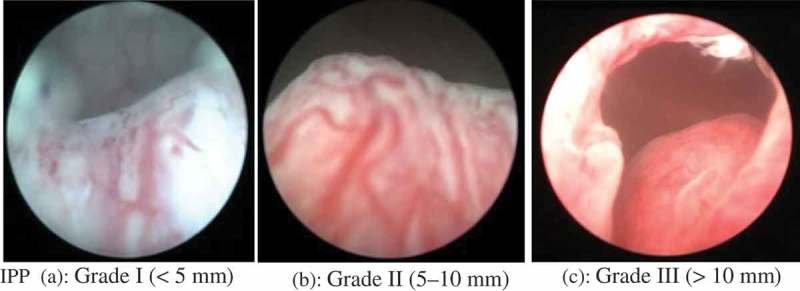
The grading system (a, b and c) for the IPP confirmed by cystoscopy. (a) IPP Grade I, <5 mm; (b) IPP Grade II, 5–10 mm; (c) IPP Grade III, >10 mm.

In all, 164 patients were selected and underwent clinical evaluation of their LUTS using the IPSS questionnaire (to evaluate voiding and storage symptoms) and a focussed urological examination. Urine analysis, serum PSA level, and renal function tests were performed, as usual. Transabdominal US (TAUS) using a Philips HD-9 machine (Philips Healthcare, Best, The Netherlands) with 5-MHz convex and linear probes and a 7.5-MHz transrectal probe was used to examine the Kidney-Ureter-Bladder region by a single operator with 10-years’ experience on a full bladder (ingestion of 1 L water over a 2-h period to achieve bladder fullness of ≥200 mL) to estimate the IPP (mm), PV (mL), and BWT (mm). IPP was measured by estimating the distance from the tip of the prostate’s protrusion in the vesical lumen to the bladder neck (Figure 1). IPP measurement was quantified and graded into three grades: IPP Grade I, <5 mm; Grade II, 5–10 mm; and Grade III, >10 mm (Figure 2). PV was determined using the prolate ellipsoid formula: (transverse diameter × anteroposterior diameter × cephalocaudal length)/2 × 100. PVR was determined after voiding with TAUS using the formula for a solid ellipse. The PV was classified as: Grade A, ≤20 mL; Grade B, >20–40 mL; and Grade C, >40 mL. A Dantec urodynamic machine (Dantec Medical Inc., Skorlunde, Denmark) was used to perform uroflowmetry and the pressure–flow UDS. Uroflowmetry was performed after calibration and the voided volume, Q_max_, average flow rate, hesitancy time, and voiding time were recorded. Men with a Q_max_ of ≥12.0 mL/s were considered to have ‘good’ flow. Using a double-lumen catheter (7–8 F) and a Duet Multi-P cystometry system (Dantec Medical Inc.) a pressure–flow evaluation was done in all patients according to the ICS recommendations. The extent of BOO was calculated using the BOO index (BOOI >40 indicates definite BOO, 20–40 is equivocal, and <20 indicates no BOO). Cystoscopy was done routinely in all patients, even though not indicated in all patients according to the standard guidelines after obtaining consent.

### Statistical analysis

The data were entered into Microsoft Excel® (Microsoft, Redmond, WA, USA) and statistical analysis was done using the IBM Statistical Package for the Social Sciences (SPSS®), version 24.0 (SPSS Inc., IBM, Chicago, IL, USA). For categorical variables, the data values are presented as numbers and percentages, and to test the association between groups the chi-squared test was used. For continuous variables, the data values are presented as mean (SD) and to test the mean difference between groups, ANOVA was used. To test for correlations between the groups, Pearson’s correlation was used. To evaluate the accuracy of IPP in identifying BOO, sensitivity analysis (receiver operating characteristic [ROC] curve) was used. All *P* < 0.05 were considered statistically significant.

## Results

**Figure 3.**
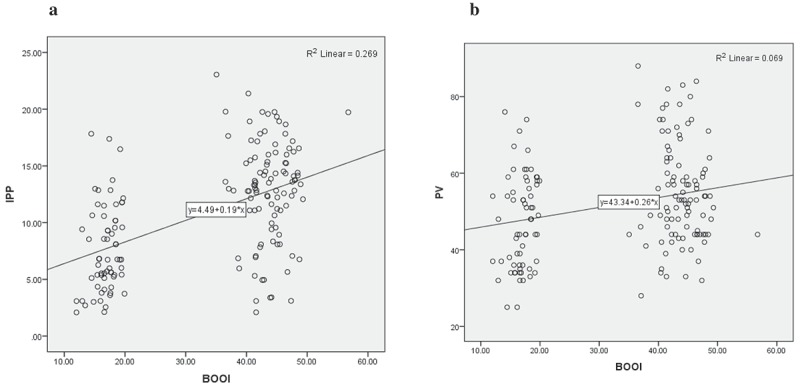
Scatter plots showing relationship between (a) BOOI and IPP (*r* = 0.586) and (b) between BOOI and PV (*r* = 0.374).

**Figure 4.**
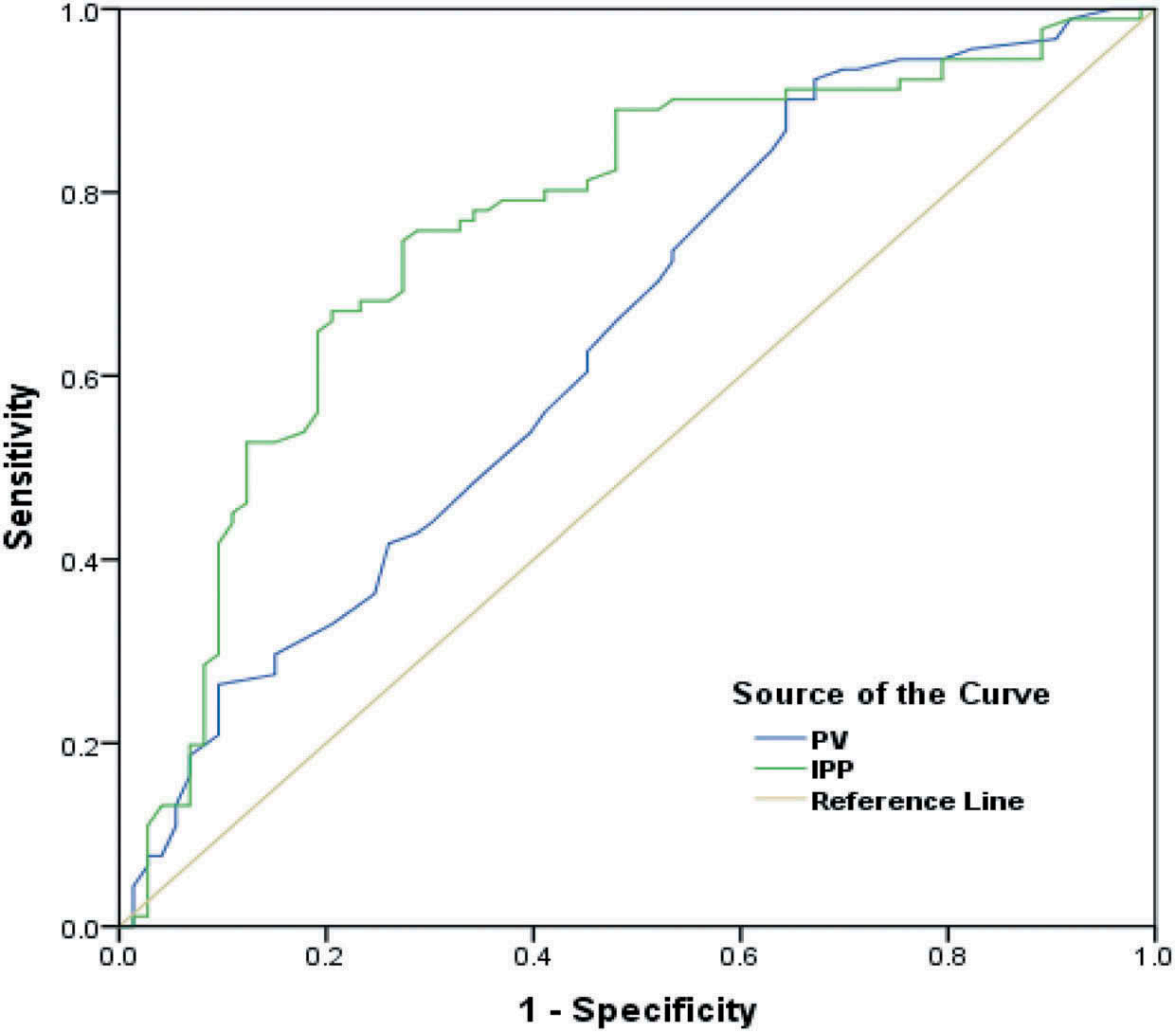
ROC curves of IPP and PV for BOO.

The mean (SD) age, IPSS, serum PSA level, and PV of the patients was 66.72 (9.88) years, 25.04 (3.34), 2.29 (1.01) ng/mL, and 51.91 (13.24) mL, respectively. The mean (SD) BWT, PVR, and IPP of the enrolled 164 patients was 5.07 (0.91) mm, 58.55 (32.88) mL, and 10.82 (4.95) mm, respectively; and the mean differences of the various clinical variables with BOO are shown in Table 1. The IPSS and QOL index had poor predictive significance for BOO (positive and negative predictive values <60%), whereas Q_max_, PVR, BWT, PV, and IPP Grade, were good predictors of BOO. When both the Q_max_ and PVR were combined as the primary evaluation for LUTS, the predictive power was less than when the IPP Grade was also included in the assessment. The mean (SD) PV in IPP Grade III [56.34 (13.33) mL] was significantly higher when compared with Grade I [48.66 (13.69) mL] and Grade II [49.24 (11.54) mL] (*P* = 0.002). The mean (SD) IPSS in IPP Grade III [26.46 (3.23)] was significantly higher than Grade II [24.57 (2.84)] and Grade I [23.46 (3.32)] (*P* < 0.001). The mean (SD) BWT of IPP Grade I [4.57 (0.97) mm] and Grade II [5.11 (0.88) mm] were significantly lower than Grade III [5.35 (0.77) mm] (*P* < 0.001). The mean (SD) IPP of IPP Grade III [13.76 (4.25) mm] was significantly higher than Grade I [3.78 (0.89) mm] and Grade II [9.73 (4.45) mm] (*P* < 0.001) (Table 1). The mean (SD) Q_max_ was significantly higher in IPP Grade I [10.31 (3.49) mL/s] and Grade II [8.46 (3.62) mL/s] than Grade III [7.29 (3.16) mL/s] (*P* < 0.001). The mean (SD) P_det_ at Q_max_ (P_det_Q_max_, cmH_2_O) was significantly lower in IPP Grade I [47.22 (18.27) cmH_2_O] and Grade II [50.85 (15.23) cmH_2_O] when compared with Grade III [66.77 (30.83) cmH_2_O] (*P* < 0.001) (Table 2). Of the 164 patients, 91 (55.49%) had BOO with a BOOI >40 (54 were IPP Grade III, 29 were Grade II, and eight Grade I) and nine (5.49%) patients had an equivocal BOOI of 20–40 [one (2.44%) IPP Grade I, three (5.17%) Grade II and five (7.69%) Grade III]. There was no BOO (BOOI <20) in 64 (39.02%) patients [32 (78.5%) IPP Grade I, 26 (44.83%) Grade II and six (9.23%) Grade III] (Table 3). The mean Q_max_ was significantly higher in IPP Grade I and Grade II than in Grade III patients (Table 4). There was a positive correlation between IPP and PV. The correlation coefficient between IPP and PV was 0.258 (*P* < 0.001). Both PV and IPP were positively correlated with BOOI. However, IPP had a better correlation (*r* = 0.586, *P* < 0.001; Figure 3(a)) than PV (*r* = 0.374, *P* = 0.001) with the BOOI (Figure 3(b)). Based on ROC area under the curve (AUC) values, the AUC for IPP was greater than the AUC for PV (0.791, *P* < 0.001 vs 0.658, *P* = 0.002) (Figure 4).

**Table 1.** Basic clinical and demographics characteristic of the patients.

	IPP Grade				
Variables, mean (SD)	I (*n* = 41)	II (*n* = 58)	III (*n* = 65)	Total (*n* = 164)	*P*
Age, years	64.05 (9.62)	66.50 (10.32)	68.60 (9.36)	66.72 (9.88)	0.067
PSA level, ng/mL	2.53 (0.93)	2.28 (0.93)	2.14 (1.11)	2.29 (1.01)	0.143
PV, mL	48.66 (13.69)	49.24 (11.54)	56.34 (13.33)	51.91 (13.24)	0.002*
IPSS	23.46 (3.32)	24.57 (2.84)	26.46 (3.23)	25.04 (3.34)	<0.001*
BWT, mm	4.57 (0.97)	5.11 (0.88)	5.35 (0.77)	5.07 (0.91)	<0.001*
PVR, mL	54.08 (35.54)	59.32 (28.44)	60.67 (35.00)	58.55 (32.88)	0.592
IPP, mm	3.78 (0.89)	9.73 (4.45)	13.76 (4.25)	9.93 (3.26)	<0.001*

*P < 0.05.

**Table 2.** Patients’ characteristics and UDS parameters based on IPP Grade.

	IPP Grade				
Variable	I (*n* = 41)	II (*n* = 58)	III (*n* = 65)	Total (*n* = 164)	*P*
Mean (SD):					
Q_max_ free, mL/s	10.31 (3.49)	8.46 (3.62)	7.29 (3.16)	8.46 (3.59)	< 0.001*
Cystometric capacity, mL	269.20 (86.59)	249.29 (75.34)	267.29 (86.18)	261.40 (82.59)	0.381
Bladder compliance, mL/cmH_2_O	32.22 (14.14)	36.47 (14.22)	31.83 (13.46)	33.57 (13.99)	0.144
P_muo_, cmH_2_O	29.40 (6.26)	33.81 (12.72)	40.19 (10.98)	35.24 (11.52)	< 0.001*
P_det_Q_max_, cmH_2_O	47.22 (18.27)	50.85 (15.23)	66.77 (30.83)	56.25 (25.05)	< 0.001*
BOOI	26.6 (11.29)	33.93 (7.99)	52.19 (14.51)	40.04 (12.26)	< 0.001*
Detrusor overactivity*, *n* (%)	3 (7.3)	7 (12.1)	34 (52.3)	44 (26.8)	<0.001**

**P_muo_**: minimal urethral opening pressure. *P < 0.05.

**Table 3.** Distribution of UDS results based on IPP.

IPP Grade	Obstruction (BOOI >40), *n* (%)	Equivocal obstruction (BOOI 20–40), *n* (%)	No obstruction (BOOI < 20), *n* (%)	Total, *n* (%)
I	8 (19.51)	1 (2.44)	32 (78.05)	41 (25.00)
II	29 (50.0)	3 (5.17)	26 (44.83)	58 (35.37)
III	54 (83.08)	5 (7.69)	6 (9.23)	65 (39.63)
Total	91 (55.49)	9 (5.49)	64 (39.02)	164 (100.0)

**Table 4.** Evaluation of accuracy of IPP in identifying BOO.

	IPP Grade II/III	IPP Grade III
Sensitivity	91.21 (83.41–96.13)	65.06 (53.81–75.20)
Specificity	45.21 (33.52–57.30)	84.93 (74.64–92.23)
Positive predictive value	67.48 (58.45–75.65)	83.08 (71.73–91.24)
Negative predictive value	80.49 (65.13–91.18)	68.13 (57.53–77.51)

Values are presented as % with 95% CI in parentheses.

## Discussion

LUTS may include voiding and/or storage urinary symptoms, and be considered a consequence of BPH. LUTS due to BPH adversely affects QOL. Usually the first-line treatment is medical, to try and relieve patients’ bothersome urinary symptoms, thus many urologists use symptoms to diagnose BPH. Although pressure–flow UDS is invasive and expensive, and is uncomfortable for patients, it is considered the ‘gold standard’ for diagnosing BPO. Several methods have been reported recently to evaluate men presenting with LUTS. The two US-derived measurements detrusor wall thickness and IPP were separately proposed as useful non-invasive parameters to predict BPO in patients with LUTS. Measurement of IPP by TAUS is non-invasive and easily reproducible, whereas patient compliance with TRUS measurement of IPP is not always good. Other non-invasive clinical variables have no significant correlation with BOO [8–10]. IPSS and PVR may reflect the severity of BOO, but the presence of bladder dysfunction has more deleterious effects [11]. Recently, several studies have reported the importance of anatomical factors in evaluating men with LUTS. Kuo [12] and Ockrim et al. [13] also proposed measuring PV and configuration, but using the transrectal approach, which might cause discomfort to patients and may not be acceptable to many men, especially when incorporated as part of the initial assessment. In our present series, prostate size and IPP were measured by TAUS, which is simple and non-invasive, and allows for evaluation of the bladder and PVR at the same time. IPP arises from the enlargement of the median and lateral lobes and causes a ball-valve type of obstruction and thus disrupts the funnelling effect of the bladder neck. The high-grade IPP group have more obstruction than the lower grade groups. Chia et al. [4] in their study correlated the degree of IPP with BPO. Of IPP Grade III cases, 94% had BOO, whilst 79% of IPP Grade I cases had no BOO on pressure–flow UDS, which correlates well with our present study. In our present study, IPP showed better correlation with IPSS than PV and there was no correlation between PV and QOL, whereas IPP and QOL correlated strongly. It has been reported that clinical parameters such as IPSS, PSA level and QOL do not correlate with BOO, which is similar to our present results [14]. Increased PV, significant IPP and PVR appeared more often in the obstructed patients and a significantly lower Q_max_ was found in obstructed patients (*P* < 0.05). Also, the present study showed significantly higher PV, PVR (*P* < 0.05) and IPP (*P* < 0.001) in obstructed patients. Keqin et al. [15] reported that the best IPP threshold was 7.5 mm (sensitivity of 75.5% and specificity of 82.6%), which was greater than our threshold of 5.5 mm. The prevailing view is that IPP correlates with BOO but does not have significant value compared with PV. The presently determined AUC value for IPP indicates that the measurement of IPP has greater diagnostic value in BOO than does PV when evaluating prostate TAUS data. BOO is dynamic and is influenced by the physical obstruction of the bladder and prostate, and we think that IPP measurement is needed in patients when PV is not excessive, and that BOO patterns may be useful. Also, IPP measurements in addition to uroflowmetry, PVR, and PSA levels likely are helpful in diagnosing BOO, in that in the present study IPP produced a larger AUC than did PSA level, Q_max_, or PVR. A recent study by Franco et al. [16] concluded that when both IPP and BWT were measured concomitantly, it had a diagnostic accuracy of 87% in detecting BOO amongst patients with symptomatic BPH. Manieri et al. [17], in their 174 patients with BPH, reported that a BWT threshold of 5 mm was best to diagnose BOO. In the present study, we demonstrated that BWT increased with IPP Grade and was an accurate predictor of BOO, with a mean (SD) threshold of 5.07 (0.91) mm, with an inverse correlation between the BWT and Q_max_. Minimal urethral opening pressure was also higher in IPP Grade III. The present study clearly points to the fact that frequent detrusor contractions during the storage phase while the bladder outlet is obstructed are presumed to increase the work load on the muscle, with consequent hypertrophy. A threshold BWT of 5 mm had 84% sensitivity and 89% specificity for detrusor overactivity. Oelke et al. [18] assessed detrusor wall thickness in patients with BOO and found a positive correlation between the degree of BOO and detrusor wall thickness, which was a more sensitive parameter for predicting BOO compared with other parameters, such as Q_max_ and PVR, and comparable with our present study. These data support the view that IPP can predict BOO, compared with Q_max_, PVR, and PV, for patients with BPH/LUTS, and may have diagnostic predictive value similar to that of pressure–flow UDS [19]. Also, by predicting BOO and defining a specific IPP threshold linked with the occurrence of BOO, we suggest that the degree of IPP can guide further treatment in patients with BPH/LUTS.

### Limitations

There are certain limitations to the present study. This was a non-randomised, unblinded prospective study without any control group. TRUS was not used to assess the chosen parameters; the present study was conducted at a single institution and included a limited number of patients. The impact of varying pre-void bladder volume on the BWT and IPP was not evaluated; however, we sought to minimise this by ensuring that enrolled patients had a pre-void bladder volume of ≥200 mL. All TAUS studies were done by a single operator, which were not re-evaluated. To date, there is no evidence to suggest whether protrusion of the prostate into the bladder is an independent factor for obstruction. While the median lobes may protrude significantly into the bladder, it is believed that the same may not be circumferentially compressive of the urethra. This may be a limitation of the present study.

## Conclusions

Although for better diagnostic accuracy a combination of investigative techniques are used, no one modality to date has been able to replace invasive pressure–flow UDS, which remains the gold standard. However, measurement of IPP with simple non-invasive TAUS is promising. In our present study, an IPP >5.5 mm was significantly associated with BOO. This knowledge should help to usefully guide the treatment of BOO in patients with BPH/LUTS.
